# Challenges and Benefits of Virtual Reality in Home-Based Poststroke Rehabilitation: Co-Design Qualitative Study

**DOI:** 10.2196/78798

**Published:** 2026-01-15

**Authors:** Trust Saidi, Ann Marie Hestetun-Mandrup, Nenad Pavel, Ingvild Kristina Hurum Rosseland, Kathinka Granum Selmer-Olsen, Nora Synnøve Mørk, Åse Bergheim, Minna Annika Pikkarainen

**Affiliations:** 1 Department of Product Design Faculty of Technology, Art and Design OsloMet – Oslo Metropolitan University Oslo Norway; 2 Department of Rehabilitation and Health Technology Faculty of Health Sciences OsloMet – Oslo Metropolitan University Oslo Norway; 3 Sunnaas Rehabilitation Hospital, Norway Bjørnemyr Norway; 4 Department of Product Design Faculty of Technology, Art and Design OsloMet – Oslo Metropolitan University OSLO Norway; 5 Forsterket rehabilitering Aker, Helseetaten Oslo Kommune, Oslo, Norway Oslo Municipality Aker Hospital Oslo Norway

**Keywords:** virtual reality, rehabilitation, people after stroke, co-design, stroke

## Abstract

**Background:**

Stroke often leads to persistent impairments that limit daily functioning and psychosocial well-being. Virtual reality (VR) has emerged as a promising adjunct in stroke rehabilitation, although research has largely focused on clinical outcomes, with limited attention to user involvement and the experiences of multiple stakeholders in the design process.

**Objective:**

This study aimed to explore the challenges and benefits of co-designing and using VR to support home-based poststroke rehabilitation.

**Methods:**

A qualitative co-design case study was applied to gain an in-depth understanding of the challenges and benefits. Rapid co-design principles were used in developing VR prototypes delivered via head-mounted displays through 3 workshops with participants with stroke, health care professionals, and VR developers from November 2023 to May 2024. A design prototype revision was done based on feedback from the workshops. Data were collected via audio-taped co-design workshops with all participants and 10 successive semistructured interviews with health care professionals and VR developers conducted in a rehabilitation hospital. A thematic analysis was performed on transcribed recordings.

**Results:**

In total, five main themes emerged: (1) adaptability for stroke-related impairments in home rehabilitation, (2) safety and ease of use, (3) goal orientation, (4) motivation, and (5) VR as a complementary tool. One of the primary challenges identified lies in the adaptability of VR systems for individuals with hemiparesis. Additionally, customization and safety concerns remain a complex barrier, as VR solutions must be capable of addressing a wide range of stroke-related impairments and aligning specific rehabilitation goals. VR demonstrated potential to enhance rehabilitation by simulating real-life tasks that encourage goal-oriented and motivating therapy. As a complementary tool, VR can enhance traditional rehabilitation by increasing the intensity and volume of therapy.

**Conclusions:**

This study offers insight into how VR can be effectively integrated into rehabilitation practices. Its integration into rehabilitation requires alignment with established therapeutic principles within VR applications, such as adjustable task-specific training and meaningful outcomes tailored to individual needs, to ensure clinical relevance and user engagement. VR should complement, rather than replace, conventional therapy by increasing training intensity, reducing therapist workload, and extending rehabilitation into the home. Thoughtful co-design with stakeholders is key to creating VR tools that bridge the gap between structured clinical care and independent recovery, offering continuous support throughout the rehabilitation process.

## Introduction

Stroke ranks as the second leading cause of death globally and is a major contributor to adults’ neurological and neuropsychological persistent impairments [1], impacting 17 million individuals annually [2]. This is a cause for concern as the demand for poststroke rehabilitation is expected to rise significantly [3], imposing considerable strains on both the quality of life of people after stroke and health care resources. Rehabilitation for people after stroke often requires a long period of rehabilitation and motor relearning [4]. Artificial intelligence (AI)–driven virtual reality (VR) solutions are expected to support individuals’ self-managed rehabilitation and have the potential to effectively engage individuals in intensive, repetitive, and task-oriented activities [5]. They can serve as useful tools for monitoring progress and enhancing decision-making in the rehabilitation process for people after stroke and health care professionals [6].

Stroke can affect individuals to varying degrees, with motor impairments being the most frequent disability. These impairments can limit motor mobility and have a negative impact on individuals’ physical activity levels and psychosocial well-being [7]. A European multicenter cohort study demonstrated that after 5 years, people after stroke experienced a decline in functional and motor outcomes, reverting to the levels observed at 2 months post stroke, highlighting that many individuals experience residual impairments [8]. Motor disabilities can vary from slight weakness to severe paralysis, impacting one’s ability to perform everyday activities like eating, cooking, and dressing independently. It is estimated that about 75% of people after stroke initially face arm impairment, with only about 50% regaining arm function within 6 months after their stroke [9,10]. Although there are several studies on the effectiveness and use of VR, with some targeting specifically upper limb rehabilitation [11-13], they are mostly systematic reviews. The empirical studies on this topic are mainly based on the evaluation of the effects of VR in the rehabilitation process and not individual experiences [14-18]. While both the systematic reviews and evaluations on the effect of VR have enhanced our understanding of the benefits of these interventions, there is a paucity of knowledge on how these positive outcomes are achieved.

Experiencing a stroke often affects more than just physical activities and functions; it also impacts cognition and personal factors. The impact on life after stroke often leaves individuals feeling vulnerable and anxious about what lies ahead [19,20]. Cognitive challenges, including difficulties with memory, attention, and problem-solving, are common, as are emotional effects, such as mood swings, depression, and anxiety [21]. VR technologies targeting cognitive function and stress management have also been found to be effective [22]; however, a recognized gap in the literature exists regarding the effect that VR has on psychological well-being, which potentially affects cognitive rehabilitation. Therefore, greater attention is needed regarding how individuals recovering from stroke engage with VR solutions. This paper seeks to contribute to the growing body of knowledge in this area. Stroke rehabilitation is often a prolonged and challenging process, requiring long-term therapeutic intervention. A study by Johansen et al [23] examining the use of VR equipment in home settings for individuals with brain injuries highlighted the ongoing need for cognitive rehabilitation following hospital discharge. Participants in the study emphasized the importance of individualized VR interventions and noted that initial training typically occurred within hospital environments. These findings suggest that exploring the early implementation of VR in controlled clinical settings, facilitated through close collaboration with health care professionals, may offer valuable insights into the feasibility and effectiveness of VR-based rehabilitation post stroke.

The gaps identified above underscore the importance of understanding how VR technology can be both designed for and experienced by users within a safe and supportive environment, one that allows for a comprehensive exploration of its potential benefits as well as its inherent challenges. This demands a focus on the process of engaging the users rather than merely evaluating the effects. Focusing on the effects without elaborating on the development process poses the danger of oversimplifying the mechanisms through which VR contributes to recovery. Without a clear understanding of the underlying processes, it becomes challenging to optimize and innovate VR interventions to maximize their rehabilitation potential. Given the limited research on the use of immersive VR for stroke rehabilitation involving clinicians, people after stroke, and VR developers, it is crucial to integrate a multidisciplinary co-design approach to ensure that clinical VR solutions are both effective and user-centered [24-26]. By involving individuals with stroke, developers, and multidisciplinary health care professionals early in the design process, we can balance desired features with evidence-based design recommendations, enhancing the relevance and usability of these digital technologies. This approach also helps prevent the development of solutions that fail to meet actual treatment needs or pose safety risks to people after stroke when using VR solutions independently at home.

Even though VR systems promise more engaging experiences, especially when it comes to repetitive movements in poststroke rehabilitation, it is still not a go-to practice for many people after stroke and therapists. This study, therefore, aims to address the identified research gaps by exploring the challenges and benefits of co-designing and using VR to support home-based poststroke rehabilitation.

## Methods

### Overview

A qualitative co-design case study was conducted to gain an in-depth understanding of the real-life phenomenon within its environmental context [27]. A single case study approach was selected as it provided a robust means to explore “how” and “why” questions within a specific context, which gave the opportunity to investigate deeper causes of the phenomenon [28]. The data were collected through different sources of semistructured interviews and workshops.

### Design Process Using Rapid Prototyping

**Figure 1:**
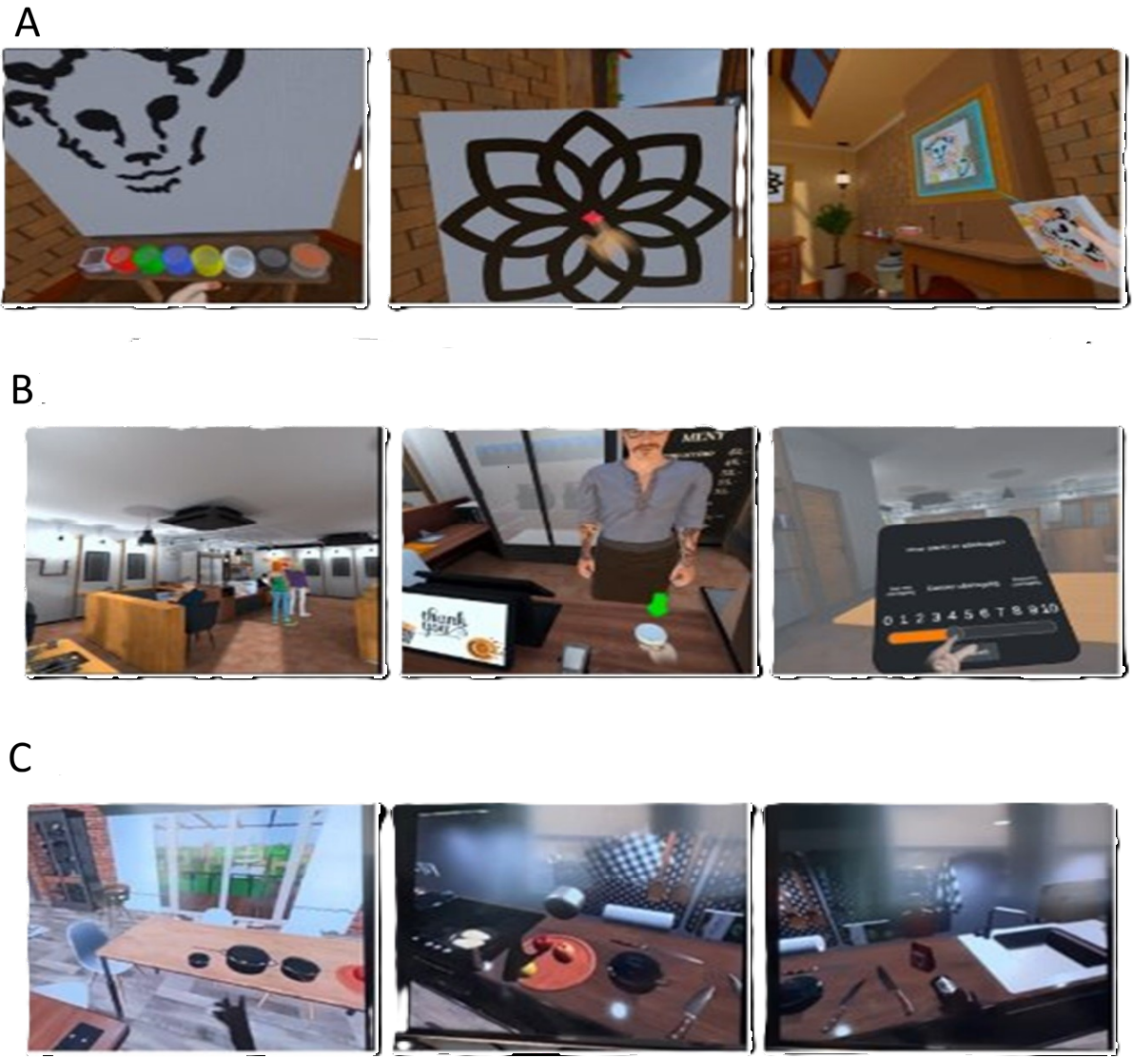
Virtual reality scenarios presented to people after stroke (n=4) during stakeholder workshops. (A) Scenario 1: Painting, (B) Scenario 2: Café, and (C) Scenario 3: Kitchen.

We applied design principles and rapid prototyping [29] to develop VR prototypes (Figure 1) through 3 workshops with a mixture of participants held between November 2023 to May 2024 with the goal of (1) understanding the challenges and map the needs of people after stroke using an existing VR café scenario; (2) discuss possibilities and evaluate features in kitchen and a painting scenarios; and (3) test, evaluate, and refine the modified prototypes of the kitchen and painting scenarios through discussions and user feedback. The prototypes were immersive VR environments delivered via head-mounted displays (Oculus Quest 3 [Reality Labs]), featuring AI-driven interactive virtual characters, while other scenarios used scripted or noninteractive virtual elements with hand tracking.

**Figure 2:**
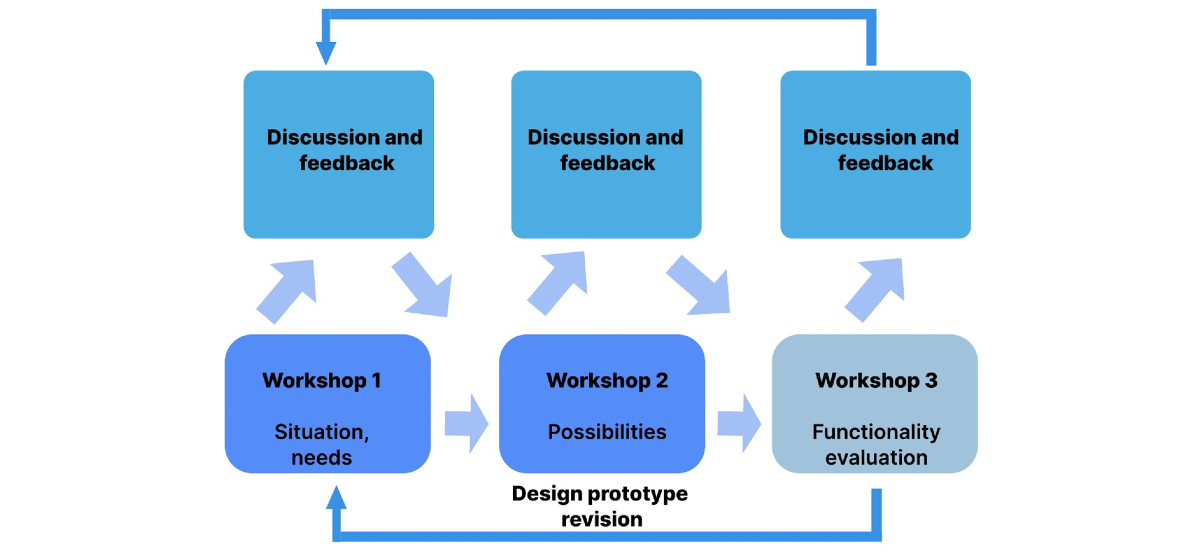
Flowchart illustrating the design workshops involving people after stroke, health care professionals, virtual reality developers, and researchers (n=16) to iteratively develop and refine a virtual reality prototype for poststroke rehabilitation.

To explore the potential of immersive environments in stroke rehabilitation, 3 VR scenarios, that is, a painting studio, a café, and a kitchen (Figure 2), were designed to simulate familiar and meaningful everyday settings. These environments were purposefully developed to promote creative engagement and functional interaction, allowing participants to practice activities reflecting real-life contexts in a safe and controlled virtual space. The 3 scenarios are illustrated in Figure 1.

The first scenario illustrates the painting environment, where the participants entered a living room and moved toward the drawing board, where equipment and colors were located. The participants could choose between freehand painting and tracing a preset illustration or figure. Guidelines for each activity were provided in all scenarios. The second scenario placed participants in a large café environment. They moved toward the cashier’s desk, ordered their food, and paid using a tablet. Participants also had the opportunity to interact with other VR avatars in the café. The third scenario illustrates a kitchen environment, where the participants could move between the kitchen counter and the dining table. They could do different activities like setting the table, placing kitchen utensils in drawers, or putting apples in a bucket.

In the first workshop, painting and café scenarios were presented to people after stroke and health care professionals. For the subsequent workshops, only painting and kitchen scenarios were further developed, as these were identified as most relevant and useful for people after stroke undergoing home rehabilitation, particularly for enhancing physical and cognitive functions. The co-design process was led by 2 researchers (MAP and NP)—a professor and an associate professor—who brought insider knowledge about the co-designing and feature-adjusting phenomena. Furthermore, 2 additional researchers (TS and AMHM), a postdoctoral and doctoral candidate, provided an outsider view to challenge assumptions. This single case approach allowed researchers to follow the co-design development process and adaptation of VR systems to the needs and demands of people after stroke, health care professionals, and VR developers.

Each workshop began with a short introduction to the aim of the VR scenarios, followed by people after stroke testing the solutions, and finally followed by 2 discussion sessions with all stakeholders and 1 exclusively with health care professionals. Feedback from the workshops informed iterative revisions of the prototype, as illustrated in Figure 2.

### Recruitment of Participants

The study was conducted in a rehabilitation hospital where participants were recruited to participate in the workshops from three distinct groups where the number of unique participants was: (1) health care professionals (n=10), (2) people after stroke (n=4), and (3) VR developers (n=2). All participants received previous written information and were invited to participate in both the workshops and follow-up interviews via phone or email. Health care professionals were recruited by the unit leader, while people after stroke were selected based on their involvement in ongoing rehabilitation programs within the hospital. The VR developers were already involved in organizing the workshops as part of their collaboration with the research team. They agreed to participate in interviews after the workshops were concluded. During the workshops, all 3 groups were asked if they were willing to participate in follow-up interviews. A total of 10 participants agreed, including health care professionals and VR developers. Additionally, 3 out of 4 participants with stroke agreed to engage in short conversations and provided feedback during VR testing. The 4 participants were recruited from a community-funded rehabilitation hospital in Norway based on the following inclusion and exclusion criteria: adults with a confirmed stroke diagnosis, speaking either English or Norwegian, and living at home in the subacute or chronic phase (more than 3 months since the last stroke) as defined by a stroke trajectory framework [30]. In addition, the participants needed to have a score of 18 or above on the Montreal Cognitive Assessment (MoCA) [31] and the ability to complete specific tasks from the Action Research Arm Test [32,33], such as pouring water from one glass to another and placing a hand on top of the head. Exclusion criteria included individuals younger than 18 years, pregnant women, and people with receptive or expressive aphasia or memory and communication impairments that hindered them from using the prototypes. The 4 participants with stroke had a balanced gender distribution and ranged in age from 53 to 64 years. The participants experienced different types of strokes; 2 experienced ischemic strokes, while the other 2 experienced hemorrhages (1 subarachnoid and 1 brainstem). None of the participants with stroke had previous experience with VR. Additionally, several health care professionals with expertise in stroke rehabilitation (ie, physiotherapists, occupational therapists, nurses, and medical doctors) and VR developers participated. The stakeholders, consisting of people after stroke, health care professionals, and VR developers, were involved in all 3 workshops as illustrated in Table 1.

**Table 1:** Overview of the participants excluding the researchers involved in the workshops (n=16) and interviews (n=10).

Stakeholder	Workshop attendance	Individual interviews
Researchers	Workshop 1: n=3 Workshop 2: n=3 Workshop 3: n=4 Total number of unique participants: n=4	—^a^
People after stroke	Workshop 1: n=3 Workshop 2: n=3 Workshop 3: n=3 Total number of unique participants: n=4	—
Health care professionals	Workshop 1: n=8 Workshop 2: n=5 Workshop 3: n=10 Total number of unique participants: n=10	Number of interviewees=8 (4 occupational therapists, 2 physiotherapists, 1 medical doctor, and 1 nurse)
VR^b^ technology developers	Workshop 1: n=2 Workshop 2: n=2 Workshop 3: n=2 Total number of unique participants: n=2	Number of interviewees=2 (VR developers)

^a^Not applicable.

^b^VR: virtual reality.

### Data Collection

Audio recordings from each of the 3- to 4-hour co-design workshops [34] and collaboratively developed individual interviews conducted at a rehabilitation hospital served as the primary data sources for this case study. A total of 10 semistructured interviews, lasting 60-90 minutes each, were conducted and audio-recorded. Open-ended questions were used in both workshops and interviews (refer to interview guide in Multimedia Appendix 1). All meetings were conducted face-to-face. Workshop 3, being both comprehensive and advanced, built upon the outcomes of Workshops 1 and 2 and served as the primary data source for this study. Both workshops and interviews were transcribed and anonymized, with no real names or personal information retained from participants, to ensure confidentiality and adhere to ethical research standards. Researchers from Oslo Metropolitan University, led by designers with experience in design-driven innovation, user experience design, and digital prototyping (MAP and NP), mediated the workshops, while clinicians provided support to participants with stroke during the testing. Interviews and initial analysis were conducted by 3 experienced qualitative researchers (AMHM, TS, and NP) from Oslo Metropolitan University, with backgrounds in physiotherapy, science and technology studies, and computer and design science. Tasks were assigned based on relevant expertise; for instance, Norwegian-language interviews were conducted by fluent researchers, while interviews with VR developers were handled by a researcher specializing in the science, technology, and society interface.

The decision to conclude data collection after 10 interviews was guided by the concept of information power [35], which asserts that sample adequacy in qualitative research depends on the relevance and richness of the data in relation to the study’s aim. Given the focused objective of gathering actionable feedback for iterative refinement of VR prototypes rather than achieving full thematic saturation, a small, purposefully selected sample was appropriate. This approach aligns with rapid prototyping methodology, which prioritizes targeted insights to inform design decisions over exhaustive theme development [36]. The study also followed participatory research principles, prioritizing a representative sample of end users to ensure relevance and applicability. The sample size (n=10) also aligns with Creswell’s [37] recommended range of 5-25 participants for phenomenological studies. Finally, triangulation [38] using audio recordings from stakeholder workshops and individual interviews enhanced the credibility and depth of the findings.

### Data Analysis

The data were transcribed verbatim using the transcription software Whisper (OpenAI) and manually checked for accuracy. Thematic analysis (TA) [39] was used to generate in-depth insights from participants’ discussions. A codebook-based TA approach, involving multiple coders (AMHM, TS, and NP), was used to enhance the credibility and consistency of the findings [40]. The primary analysis was conducted by 3 researchers, all of whom were experienced in conducting qualitative research and applying TA as an analytical method. Further analysis was conducted collaboratively by all authors of the study and validated by health care professionals to ensure the study addressed both theoretical and clinical aspects. The analysis followed Braun and Clarke’s 6-phase framework, beginning with familiarization through repeated readings of the data to gain a comprehensive understanding. Subsequently, inductive and descriptive codes were applied, which were then grouped into preliminary themes. These initial themes were collaboratively reviewed and refined by the research team to ensure consensus about their accuracy and reliability. The processes of defining, naming the themes, and reporting the findings were conducted collectively.

To illustrate the development of the theme “safety and ease of use” using Braun and Clarke’s 6-phase framework, we present an example from the analysis process. In the familiarization phase, repeated readings of transcripts revealed frequent concerns about physical safety, navigation, and psychological comfort in VR. Initial coding grouped statements about uncertainty, environmental awareness, and support needs under relevant codes, which were later collated into the broader theme. In the reviewing phase, we ensured this theme was coherent and differentiated from other related themes like “adaptability for stroke-related impairments,” which focused more on task customization. “Safety and ease of use” emphasized risk minimization and user confidence. During theme definition and naming, we finalized it to reflect participants’ emphasis on secure, intuitive, and supportive VR environments. Final reporting included selecting illustrative quotes to demonstrate how safety concerns informed both design recommendations and user engagement.

### Rigor

Rigor was obtained through frequent debriefing and discussions among coders during data analysis and collaborative theme development within the research team. The qualitative data obtained from audio recordings of the workshops and individual interviews enabled triangulation and cross-references between findings [40], which strengthened the credibility of the results. The theoretical flexibility that TA offers was well-suited to accommodate the diverse perspectives and knowledge production of the multidisciplinary research team [41], which in this study included specialists in allied health, computer science, and design.

### Ethical Considerations

The Norwegian National Research Ethics Committee for Medical and Health Research assessed the study and determined the need for ethical approval. They declared that this study’s focus on health service research fell outside the scope of the Health Research Act § 2 and therefore did not require approval (ref 651236). Instead, this study was approved by the Norwegian Agency for Shared Services in Education and Research (ref 857865) to ensure compliance with privacy protection regulations. Participants received both oral and written information, and informed consent was obtained before the workshops and individual interviews. To protect the privacy of the participants, the study data were deidentified by using pseudonyms for all participants. Participants did not receive any financial or material compensation for their participation.

## Results

### Overview

Different participants with stroke, multidisciplinary health care professionals, VR developers, and researchers were present and participated in all workshops. There was a variety among the stroke individuals who were engaging in their first, second, or third workshop with the VR scenarios. Although engaging with avatars and objects in the café scenario was engaging, the stakeholders believed it did not target the rehabilitation principles. In the second workshop, this was amended by engaging in the kitchen and painting scenario. In the third workshop, these 2 prototypes were further developed by gamifying certain arm movements, targeting the affected arm in particular. The following results are based on the participants’ needs and key challenges and benefits that were generated in workshops 1 and 2, and applied in workshop 3, which form the bases of this study, together with retrospective individual interviews with health care professionals. The findings generated five themes: (1) adaptability to accommodate stroke-related impairments in home rehabilitation, (2) safety and ease of use as fundamental in VR stroke rehabilitation, (3) goal orientation, (4) motivation, and (5) VR as a complementary tool.

### Adaptability to Accommodate Stroke-Related Impairments in Home Rehabilitation

Participants viewed VR as a promising tool for poststroke home rehabilitation, offering interactive environments to aid motor recovery. However, they stressed that its usability depended on adapting to stroke-related impairments, such as accommodating both right- and left-handed hemiparetic users, as the first workshop primarily targeted right-hand users.

Customization was highlighted as crucial to address diverse needs, including issues like putting on VR glasses, gripping tasks, and aligning activities with rehabilitation principles. Participants stressed the importance of personalizing rehabilitation, as each person after a stroke faces unique challenges. Effective poststroke rehabilitation requires balancing engagement and endurance, as cognitive load and fatigue often limit people after a stroke. VR therapy demands motor and mental skills, making it vital to tailor intensity and duration to each individual’s ability. It was noted that VR could overwhelm people after stroke, limiting its effectiveness.

When it comes to effectiveness, dosage and repetitions, VR will take more mental capacity because you use cognitive functions alongside motor function. Many people with stroke have fatigue; some can only do it for 10 minutes before their battery is low.

Karla

The potential for AI-powered VR solutions to enhance customization was discussed, with many participants emphasizing the rehabilitation goal of fostering independence and regaining agency. However, participants cautioned that VR was not suitable for everyone. Discrepancies between VR command techniques of gripping and actual abilities for people after stroke emerged during testing:

Therapist: You can also try to lift the kettle and the red bag there.

Person after stroke, first-time user: I try with the left hand first.

Person after stroke, first-time user: Just press through, sort of?

VR developer: Yes. Use the whole thing. I had to practice a bit with gripping, because I thought I could grip with my fingers.

VR developer: You have to use your whole hand, sort of, to grip.

Therapist: Then you try to open your hand, and then... Like that, yes!

Person after stroke, first-time user: Incredible.

Participants also noted errors between VR scenarios and real-world tasks. One participant described challenges with gripping:

You can pick up things and put them in a drawer, but it’s hard to grip them. Then it kind of falls and suddenly pops up again...You haven’t managed to do what you intended...Maybe the tasks are too difficult or not well adapted.

Jane

Additional challenges included the difficulty of putting on heavy headsets for those with reduced arm function or configuring the VR system. One participant emphasized the need for customized adjustments to accommodate for stroke-related individual differences and goals:

I believe that those with severe cognitive impairments or difficulties with visual mapping might find VR too difficult initially. If we could personalize this, because one person’s goal could be the opposite to others.

Sue

Participants recognized that VR required a certain level of cognitive function. Practicing new strategies in advanced tasks like kitchen training posed greater challenges than simpler tasks focused on errorless learning, such as dressing. Familiar settings were suggested to enhance engagement and skill transfer from virtual practice to real-world tasks. While cognitive impairments such as visual mapping and adjusting to new surroundings could cause difficulties, VR’s potential to shift focus away from disability and reduce learned nonuse was noted. One participant explained:

When you enter that room, you slightly forget what is affected. And when you don’t see that the arm isn’t working, perhaps it will engage a bit more.

Betty

Kitchen training was widely regarded as a familiar and widely used element in rehabilitation. Participants noted that a VR kitchen could provide additional practice opportunities, as kitchens are a universally relevant and accessible setting for most people after stroke. A participant emphasized the importance of adjustable environments to accommodate individual needs.

“*You can put things high and low. You have the ability to increase or decrease the height of your entire kitchen, the cabinet, bench, whatever, so you can lower it or increase it for adjusting the difficulty level for your needs but also making it easier to use or potentially putting it to the exact same height as you have everything at home.* [John]

He also stressed the need for customizable difficulty levels, such as easy, medium, and hard, while acknowledging that further customizations were necessary to address individual differences. Many participants agreed that a one-size-fits-all approach would not work, but segmenting recovery stages into acute, subacute, and chronic phases, or tailoring tasks based on gripping ability, was proposed as a viable strategy.

### Safety and Ease of Use as Fundamentals in VR Stroke Rehabilitation

Ensuring safety was a fundamental consideration in VR-based stroke rehabilitation. Many people after stroke face mobility impairments, balance issues, and cognitive challenges, increasing the risk of falls or disorientation in virtual environments. During the workshops, one health care professional highlighted their obligation to people after stroke:

We cannot tell them they can do it alone if we are not sure that it is safe.

Jane

Participants noted that a secure VR environment could encourage engagement and push physical limits, enhancing rehabilitation progress. However, some participants warned that VR might create a false sense of safety. As one participant with stroke explained:

Now I can’t see where my legs are or anything. It’s an uncertainty. I feel like going over to hold onto the table, but that won’t work. Now I’m trying to do as I do at home and park the walker next to the kitchen door.

Person after stroke, second-time user

The transition from institutional rehabilitation to home-based therapy was identified as a critical phase, often accompanied by anxiety and uncertainty. One participant noted that VR could reduce anxiety and prevent early readmission to health care facilities:

We have to make sure they go home without as much anxiety, because when they do, they might just return early to the healthcare system due to a fall or stress from something minor. With the help of virtual reality, we could avoid this because we've tested it already at “home”, but at the institution.

Tom

Participants emphasized the need to carefully consider the difference between VR tasks and real-world activities. He further explained:

That’s going to be challenging, since there is a clear difference between how difficult something will be in VR versus in the real world, like cutting vegetables with a knife.

Tom

Some participants noted that successfully completing tasks in VR but failing in real life could lead to disappointment, especially when tasks required a higher degree of task complexity.

One highlighted the importance of minimizing unnecessary movement in home-based VR rehabilitation:

If someone is going to use it alone, I would limit tasks where you have to walk far, because I wouldn’t consider that safe.

Jane

Similarly, Kate pointed out that while real-life practice is ideal, VR enables people after stroke to safely train in everyday activities like walking, empowering them to actively engage in their recovery.

Beyond physical safety, participants also discussed the psychological benefits of VR, particularly its ability to reduce the fear of failure, which often prevents people after stroke from participating in rehabilitation activities. Ben underscored the importance of structured support for VR in both institutional and home settings:

Using it in our hospital department would be great because they can use it in their rooms with instructions to be seated during activities. And it can be used in the later afternoon when they’re done with daily [therapy-assisted] rehabilitation training. At home, they would be instructed to sit in a chair or a sofa with a space around them in case they get excited and get up.

Ben

The focus on sitting exercises typically placed occupational therapists in charge of VR rehabilitation due to traditional task distribution. However, health care professionals raised concerns about the safety and feasibility of using the VR system without therapist assistance, questioning whether users could operate and administer the system without assistance. Technological advancement, such as developing hybrid and mixed reality VR rooms, were proposed as potential solutions to address safety concerns. One participant with stroke explained:

I would need the ability to sense the outline of it [the walker], because it is safer when I know where it is. Otherwise, I could have practiced in the real kitchen, so it was fairly similar.

Person after stroke, second-time user

Furthermore, the presence of a caregiver or therapist was seen as essential for building user confidence in using VR, as indicated by Maud:

It’s very important to have someone who watches what you and [make sure] you do it carefully. If you fall, there’s someone with you, and you feel secure. Our people with stroke have belts on, and the physio holds the belts from the back. Maybe these people with stroke can’t walk without a stick, but with that support, they feel very secure.

Maud

One proposed solution, which came up during workshops, was to differentiate between safer, low-scale tasks for home use and more challenging tasks, such as standing or increased reaching activities, to be conducted during follow-up sessions at the hospital.

### Goal Orientation

The participants highlighted that a goal-oriented approach was essential in poststroke rehabilitation, as it helped people stay motivated and engaged after stroke. VR was perceived as a platform to structure rehabilitation programs around individualized goals, allowing people after stroke to track progress and experience achievement. The participants found that incorporating meaningful, measurable objectives encouraged consistent participation and reinforced the benefits of repetitive practice. Observing progress and perceiving tangible improvements increased commitment to rehabilitation exercises. Several participants stressed the importance of person-centered VR rehabilitation, where goals align with daily needs and personal aspirations. Karla noted:

The person with stroke’s goal is everything in the rehabilitation. If someone wants to dress independently, we have to focus on that. The person with stroke is in the driver’s seat.

Karla

Participants emphasized that VR technology should focus on customization in goal setting to enable people after stroke to work on relevant activities, like regaining independence in dressing, cooking, or mobility. To maximize effectiveness, VR interventions needed a clear focus.

I think it’s really important if you use this in a rehabilitation program, you need to specify your target. Is it cognition, arm movement, or balance?

Tom

However, limitations were noted where VR could not adapt to all needs. During the workshops, a health care professional explained:

Some patients can only use this movement—just this—but others need to grip something. Then, the patients need resistance, and this [VR] won’t improve their function like the real activity. If they are going to move the paint, there is some weight in the paint, and you dont get transferability. To see improvement at home, they must do it similarly.

Physiotherapist, Workshop 3

Participants suggested that providing multiple training options would enhance adherence and long-term commitment to therapy. Effective VR rehabilitation required targeting more tasks that mirror real-life activities to develop skills essential for independent living after discharge, such as putting up a painting, washing the dishes, or putting on clothes. It was important that the chores or activities used in rehabilitation served a specific therapeutic purpose. Kate pointed out:

An activity like painting, you don’t want to be a better painter, but you want your arm to get better. That’s the method.

Kate

From a therapist's perspective, this highlights how everyday tasks can be repurposed as meaningful rehabilitation goals, where the focus is on physical recovery rather than mastering the activity itself. A blended approach, where people after stroke first practiced activities in VR and then transferred skills to real-life settings, was suggested to reinforce motor learning and recovery. Repeating engagement in virtual tasks before real-life performance helped internalize movements, build confidence, and strengthen neural connections essential for motor function.

However, not all participants’ goals were covered by VR prototypes. One participant with stroke noted:

There is no gait training, at least not intentionally. I would have to be on a treadmill, walking into the woods. This is for the arm, though.

Person after stroke, second-time user

Tracking progress in VR was seen as a powerful motivator for people after stroke. Participants explained that observing measurable improvements encouraged continued training and inspired confidence to tackle more demanding tasks. They emphasized that VR should serve as a stepping stone, bridging the gap between virtual practice and real-life application. However, there was a need for a clear focus on whether VR training aimed for improvement or sustainability. Gamification elements, like points or rewards, were considered ineffective unless the real-world relevance of the tasks was clear for people after stroke:

It’s not necessarily the case that you get motivated by getting extra points, if you don’t quite understand why you’re doing this.

Jane

Ensuring VR-based rehabilitation included familiar, everyday tasks enabled explicit goals in a more engaging, functional, and transferable home environment. Therefore, a variety of VR rehabilitation activities, such as hanging clothes in the laundry room or other meaningful daily activities, were considered valuable.

### Motivation

The participants emphasized that motivation played a crucial role in the effectiveness of VR rehabilitation for people post stroke, ensuring long-term engagement with their rehabilitation program. Tom noted the importance of regular follow-up sessions for maintaining motivation.

We can see when they were very motivated here. They go home, and there are only challenges, no solutions. But when they return for follow-up sessions, they’re extremely motivated.

Tom

Motivated individuals were more likely to push themselves and put in the effort required to achieve their rehabilitation goals, potentially leading to better motor outcomes and quality of life. People with stroke noted that the VR scenarios stimulated learning and movement, enabling them to train without being consciously aware of it.

Person after stroke, third-time user: Now I have used the entire room, I see.

Therapist: Now we have followed you around the entire room.

Person after stroke, third-time user: Today’s workout.

VR developer: Shall we see how many points there have been. It sounded like it was beeping a lot.

Karla highlighted how competition could drive people after stroke to improve their performance.

It’s motivating when it’s like a competition. You did this last time, now try to reach this one for the progression of function.

Karla

Setting and achieving progressively challenging goals allowed people after stroke to experience significant functional gains, reinforcing their overall commitment to recovery and reaching rehabilitation outcomes. One participant noted the extrinsic motivation provided by VR and its effects on the brain.

Because when you do that, your brain tells you it’s very useful, because you get points.

Person after stroke, second-time user

While the rehabilitation process could be challenging and frustrating, motivation was seen as a driver to overcome the physical and emotional barriers, persisting through difficulties, and maintaining a positive attitude toward recovery. Many participants emphasized the role of human support in maintaining motivation, as Ben explained:

If you’re not motivated or very depressed, your life is over. We won’t do anything with you. You need humans to train with you.

Ben

Health care professionals’ emotional and psychological encouragement was regarded as crucial in helping people after stroke stay engaged. Motivated individuals were more likely to communicate their preferences and needs, allowing for a more personalized and active rehabilitation experience.

In addition, motivation was closely linked to psychological well-being. Engaging in meaningful and enjoyable activities improved mood, reduced anxiety, and enhanced overall mental health, which was crucial for recovery. Sue underscored the role of curiosity in maintaining motivation.

It’s more like an opportunity to explore and be curious. If you manage to get curious, you have less capability to be afraid or be sceptical.

Sue

Building confidence was another key factor in motivating people after stroke to embrace new challenges and persist with VR. One suggested strategy to foster confidence was visual progress tracking.

If you could implement that, for example, in painting stations, storing your paintings so you could see at the beginning I was able to make a line that was so big, but after two weeks I’m able to make a line that’s this big, could be a motivational tool.

Sue

Seeing tangible improvements over time reinforced the belief of people after stroke in their abilities, motivating continued rehabilitation efforts. Jane emphasized the importance of simplicity and ease of use for maintaining motivation.

For people with stroke to use it themselves, they must have high motivation. It must be simple enough to master quickly, not something they struggle with and give up in the end. It must be meaningful and beneficial for them.

Jane

Others suggested integrating VR with existing digital training programs, such as Exorlive, to offer a self-training option to therapy-assisted activity training. Ben highlighted the difference in outcomes between motivated and unmotivated individuals.

If you’re a person who wants to do better, you have a very clear goal. You just do that with the help of others or sometime without any help. But if you’re a person like, oh, now this is my life, I’m going to live with it, and you’re not motivated to do anything, you might just sit there and let it go day by day.

Ben

Those with clear goals and motivation were likely to strive for improvement, while those without motivation might become complacent.

Finally, John emphasized the importance of creating varied and personalized scenarios to maintain motivation, stating:

To motivate people, it would be more interesting to create different environments, such as a bedroom where you can dress yourself or a garage where you can tinker and build with your hands.

John

By providing scenarios aligned with the specific goals and interests of people after strokes, the rehabilitation process becomes more rewarding and engaging, fostering long-term motivation.

### VR as a Complementary Tool

VR was seen as a valuable complement to stroke rehabilitation, enhancing training intensity and offering structured exercises in both clinical and home settings. While not a replacement for traditional therapy, it effectively supported recovery by supplementing existing approaches. Given the limited window for optimal recovery, its role in maximizing progress was emphasized. Mary highlighted VR’s contribution to intensive training.

Our institution specializes in giving intensive training. I don’t think VR could match that, but as an additional supplement it would be amazing. Together we could help people with stroke recover more quickly. Especially for people with stroke, there is a time period to get as good as they can get.

Mary

This highlights that VR should complement, not replace, therapist-led rehabilitation by enhancing therapy intensity during the critical recovery phase. Additionally, participants proposed implementing validated clinical measurements, goal assessment scales, or kinematic data to monitor outcomes and progress within the VR system during the workshops. Betty noted the alignment between the painting scenario and existing cognitive tests, such as MoCA, suggesting VR’s potential for cognitive diagnostics in addition to general executive functions:

Because we have a test where we follow a line with a pen from number to number, or from numbers to letters. That kind of cognitive test. So, both for cognition and physical aspects, it [VR] is, if I may say so, very useful.

Betty

Individuals with stroke face unique challenges, requiring personalized rehabilitation plans tailored to their specific needs. VR adaptability offers a promising tool for delivering customized training that complements traditional therapy, ensuring alignment with each person’s circumstances. VR was seen as a tool to bridge this gap by providing structured exercises that encourage movement and engagement.

It’s another intervention in addition to what we do. I think it would increase the activity for the people with stroke to use their arm more than they otherwise would, if they have such an aid in addition to a therapist.

Jane

During workshops, VR was seen as a controlled environment where people after stroke could practice real-life tasks safely before transitioning to real-world applications. Jane highlighted this advantage:

We’ve discussed it being a supplement to training and in a safer setting for the people with stroke. It will not be instead of, but it may increase the activity between each session. Someone has concrete things to practice until next time, then practice in a real kitchen situation when someone is present to secure the situation.

Jane

Combining home-based VR training with in-person therapy was considered an ideal model for stroke rehabilitation. Participants, like Kate, emphasized that while VR enabled frequent, self-paced training at home, regular sessions with a therapist were essential to maintain motivation. This hybrid approach demonstrated VR’s potential to extend rehabilitation beyond clinical settings while preserving professional oversight. Balancing self-practice with therapist support was considered crucial. However, as Betty noted, the attitude and behavior of staff introducing VR could significantly influence patient engagement.

Let’s say the therapist has zero interest in VR. That could easily rub off on the patient. (..) I think it makes a big difference who introduces it to the patients.

Betty

As stroke rehabilitation requires extensive repetition to regain lost motor functions, VR was seen as an efficient way to deliver high-volume training, ensuring people after stroke get the practice needed for meaningful improvement. One participant explicitly noted they lacked solutions targeting volume training:

The advantage of VR is that you can do high-volume training efficiently. In rehabilitation of people with stroke, you need to spend hundreds of training hours to achieve some really big goals.

John

VR was seen as a possible way to make repetitive training more accessible and help people commit to the long-term rehabilitation process necessary for significant recovery. While VR was valuable, it was perceived as a stepping stone for real-life training. During the second workshop, an occupational therapist participant emphasized the limitations of VR in meeting diverse patient needs, noting that some individuals require functional resistance and weight-bearing activities to achieve transferability to real-life tasks:

Most people understand that VR is a supplementary tool along the way that bridges the gap, making it easier, but they know they need real life [activities] because that’s where they are anyway.

These results show that VR should not replace traditional therapy but serve as a tool to facilitate a smoother transition to functional activities.

### Summary of the Key Findings

The integration of VR into stroke home rehabilitation presents both significant challenges and promising benefits. One of the primary challenges lies in the adaptability of VR systems for individuals with hemiparesis, as these systems must accommodate both right- and left-handed users. This requires considerable technical resources and expertise to ensure that the technology can be effectively tailored to the diverse needs of people after stroke. Additionally, customization remains a complex barrier, as VR solutions must be capable of addressing a wide range of stroke-related impairments and aligning with specific rehabilitation goals, which can vary greatly from person to person. Another critical concern is the balance between engagement and endurance. While VR has the potential to make rehabilitation more stimulating, it is essential to calibrate the intensity and duration of exercises to prevent cognitive overload and fatigue, which are common among individuals with stroke. Usability also poses a significant hurdle; many people after stroke experience limited motor function, which can make it difficult to interact with VR headsets and systems. Furthermore, challenges in visual mapping and task comprehension can hinder the effectiveness of VR-based interventions.

Despite these challenges, VR offers several compelling benefits for enhancing stroke rehabilitation at home. The integration of AI into VR platforms can enable highly personalized rehabilitation experiences, adapting in real time to the user’s progress and specific needs. This level of customization can significantly enhance the efficacy of home-based therapy. Moreover, VR has the potential to increase motivation and engagement through gamification, progress tracking, and the simulation of meaningful, real-life tasks that resonate with users. VR also provides a controlled and safe environment for practicing daily activities, which can help reduce anxiety and better prepare people after stroke for real-world challenges. As a supplementary tool, VR can enhance traditional rehabilitation by increasing the intensity and volume of therapy. This aligns with 10 key principles of experience-dependent neuroplasticity, where factors such as specificity, high-repetition, intensity of stimulation, timing, task-specific training, auditory stimuli, and behavioral experience are essential for optimizing recovery outcomes after brain damage [42]. Textbox 1 provides an overview of the main challenges and potential benefits associated with using virtual reality in poststroke rehabilitation. It highlights critical considerations for implementation and the opportunities VR offers to enhance recovery outcomes.

**Summary of the key challenges and benefits in the use of VR for rehabilitation of people after stroke.**

**Key challenges in virtual reality (VR) usage for people after stroke rehabilitation**

- Adaptability for hemiparesis
- VR systems need to accommodate both right- and left-handed users, which requires significant resources and skill to adapt to various people after stroke needs.
- Customization barriers
- VR technology must address individual stroke-related impairments and specific rehabilitation goals, which can be complex and demanding.
- Balance between engagement and endurance
- Tailoring the intensity and duration of VR exercises to avoid overwhelming people after strokes is crucial, especially considering cognitive load and fatigue.
- Usability concerns
- VR headsets and systems can be challenging for people after strokes with limited motor function, and difficulties in visual mapping and understanding tasks.

**Key benefits in VR usage for people after stroke rehabilitation**

- Enhanced customization with artificial intelligence
- Artificial intelligence–powered VR solutions can provide personalized rehabilitation experiences, address individual needs, and enhance home-based therapy.
- Motivation and engagement
- VR can make rehabilitation more engaging through gamification, tracking progress, and providing meaningful, real-life tasks that motivate people after strokes.
- Controlled and safe environment
- VR offers a controlled environment for practicing real-life tasks safely, reducing anxiety and preparing people with strokes for real-world challenges.
- Supplement to intensive training
- As a complementary tool, VR can increase therapy intensity, providing high-volume, repetitive training efficiently to maximize recovery outcomes.

## Discussion

### Principal Findings

This study explored the challenges and benefits of co-designing and using VR to support home-based poststroke rehabilitation, with a particular emphasis on user involvement and experience. The TA revealed 5 key themes that offer insight into how VR can be effectively integrated into rehabilitation practices. Adaptability emerged as a central concern, highlighting the need for VR systems to accommodate a wide range of stroke-related impairments, including motor, cognitive, and sensory limitations. Participants emphasized the importance of customizable interfaces and adjustable task difficulty to ensure accessibility and relevance. Safety and ease of use were also critical, with users expressing a preference for intuitive designs that minimize physical strain and cognitive overload, especially in unsupervised home settings. The theme of goal orientation underscored the value of VR in facilitating task-specific training that aligns with personal rehabilitation goals, thereby enhancing the perceived relevance and purpose of exercises. Motivation was closely linked to the immersive and interactive nature of VR, which participants found to be more engaging than traditional methods, potentially supporting persistent participation over time. Finally, the role of VR as a complementary tool reflected a shared understanding that VR should not replace conventional therapy, but rather augment it, offering additional benefits for practice and reinforcement in a flexible, home-based format.

The study has revealed that one of the primary benefits lies in the adaptability and individualization of VR environments to meet the diverse needs of people after stroke. As noted in the study, many people after stroke experience hemiparesis, necessitating VR systems that accommodate both right- and left-affected users. It was noted that the final prototype involving the kitchen scenario factored this in by rewarding participants for using their affected arm. The ability to individualize VR experiences to address specific activities impaired by stroke, such as initially setting up the VR system and further conducting activities, such as reaching and gripping objects, is critical for effective rehabilitation. With appropriate individualization, VR systems have demonstrated significant effectiveness in improving various aspects of stroke rehabilitation, including upper limb function, cognitive abilities, and balance [25,43,44].

However, to bridge the gaps between the recovery process in stroke rehabilitation, technology, and clinical practice, an awareness, exploration, experimentation, and evaluation framework ensuring the solutions are engaging, accessible, accountable, and adaptable is advocated [45]. A common challenge with commercially available gaming technology, such as VR, is that even though it provides immersive, engaging, and tailored therapy (accessibility and engagement), it is not easily adapted for people after stroke, especially those experiencing limited movement or cognition. Based on our assessment, we argue that the complexity of the devices makes them difficult to adjust, posing a challenge for people after stroke to secure them properly and customize them to their individual needs. Although customization was frequently proposed to meet the individual needs of users, this highlights a critical tension. In practice, effective customization demands considerable resources to adequately and efficiently respond to the diverse and complex needs of individuals living with stroke. As indicated by one study, reducing the complexity to one working application on the goggles can limit potential errors [23]. In this study, some individuals required additional assistance in adjusting the headset or coping with cognitive impairments. There is a paradox between the ideal of customization and the reality of implementing it effectively. Achieving this may involve a balance between providing customized opportunities and still making it feasible to implement and ensure its ease of use by categorizing similar users into groups.

VR has the benefit of providing multiple training environments that target individuals by engaging them in meaningful and enjoyable activities, as particularly found in the kitchen scenario. Functional exercises, such as washing dishes, dressing, and brushing teeth, allow people after stroke to develop skills essential for independent living after discharge [46]. This aligns with one study [47], in which people after stroke found different activities in everyday life easier to perform since starting their VR training, such as opening a drawer or applying toothpaste to a toothbrush. Enriched environments are essential in leveraging VR for stroke rehabilitation, as they offer diverse and stimulating approaches to problem-solving and skill development [48]. Some overarching principles that guide an enriched environment approach are complexity, variety, and novelty of the environment as well as targeting underlying needs [49]. In our study, the VR scenarios targeted all principles, but ideas for scaffolding complexity and variety tailored for each individual were proposed.

By simulating real-life scenarios and interactive challenges, VR provides people after stroke with engaging and repetitive practice, which is crucial for neuroplasticity and motor recovery [50]. Additionally, the adaptability of VR allows therapists to modify the learning environment to match individual needs, for example, by adjusting the levels of difficulty, thereby fostering a more immersive and intensive experience that enhances engagement and accelerates rehabilitation progress. This flexibility not only improves functional recovery but also increases motivation and adherence to therapy, making VR a promising tool in modern rehabilitation strategies [51]. In a study by Gustavsson et al [47], VR provided a feeling of being in a different world where users felt they were reaching higher and moving faster. However, VR environments can deceive the mind into believing they are a real-life environment. This illusion is a cause for concern, as the immersive nature of VR can make the brain perceive the simulated surroundings as authentic, thereby inducing a false sense of safety and achievement [52].

Given the challenging nature of rehabilitation for individuals who have experienced a stroke, VR plays a critical role in motivating them by ensuring their active participation and long-term effort in the recovery process. Engaged people after stroke are more likely to adhere to their therapy schedules and participate actively in their rehabilitation exercises. Rehabilitation can be challenging and frustrating, and motivation helps people after stroke overcome these barriers, persist through difficulties, and maintain a positive attitude toward their recovery [25]. It drives users to push through the physical and emotional challenges of rehabilitation, helping them to stay committed to their therapy even when progress seems slow or when they encounter setbacks. By fostering a sense of achievement and progress, motivation can transform the rehabilitation experience from a daunting task into a rewarding journey.

However, maintaining motivation can be difficult, especially for people after stroke facing mental health challenges, such as depression or anxiety. These conditions can significantly impact their willingness to engage in rehabilitation activities. While we saw that the use of gamification elements, such as points and rewards, served as incentives to motivate people after stroke, they work only if people after stroke understand the real-world relevance of their tasks. Without this understanding, the motivational impact of these gamification elements may be significantly diminished. This highlights the need to design VR programs that closely mimic real-world scenarios to maximize their effectiveness.

Another benefit of VR is that it creates a controlled and secure environment that encourages people after stroke to engage more actively in rehabilitation by reducing the fear of injury, thereby promoting better recovery outcomes when they feel safe enough to push their physical limits. However, this is contingent on structured supervision and support, which are essential to mitigating risks and enhancing user confidence, particularly given the mobility impairments, balance issues, and cognitive challenges faced by people after stroke, which increase the risk of falls or disorientation [25,44]. The importance of therapist involvement in tutorials and adjusting difficulty levels when engaging with VR is highlighted in multiple studies [23,47]. This is particularly important for individuals recovering from stroke, as prolonged exposure to VR may induce motion sickness or dizziness. Additionally, those with impairments in executive functioning and problem-solving challenges observed during our workshops may find it especially difficult to navigate and adapt to such technologies. To maximize the benefits of VR therapy, careful system design, real-time monitoring, and gradual progression in exercise intensity are essential to ensuring a safe and effective rehabilitation experience [53].

While support and guidance in the use of VR emerged as indispensable, this could present challenges in terms of manpower due to the decreasing number of health care professionals. The presence of therapists significantly motivated many individuals with stroke to continue engaging with VR activities. However, this reliance on external motivation could become problematic once they are discharged home, where self-motivation is essential for ongoing rehabilitation [54]. It is not feasible to maintain this level of support when individuals with stroke transition to their home setting. Many people with brain injuries are dependent on help from their families when using digital technologies, such as VR, at home [23,54].

The use of VR presented an advantage in facilitating a goal-oriented approach by enhancing consistent participation and reinforcing the benefits of repetitive practice. The application of motor learning principles in VR design is advocated to enhance rehabilitation outcomes [25]. This includes progressively challenging tasks, real-time performance feedback, and multimodal feedback [25,43]. The development process through the workshops led to improvements in VR content in line with standardized stroke guidelines, such as goal- and task-specific activities and quantifying activities. However, it emerged from the study that the challenge lies in ensuring that VR interventions are carefully designed with clear and specific goals, whether to improve cognitive function, enhance arm movement, or restore balance. It is important that the rehabilitation goals are aligned with the daily needs and personal aspirations of the people after stroke. This requires a deep understanding of each person’s lifestyle, preferences, and long-term objectives. Achieving this alignment is challenging, as the goals can change over time, requiring continuous assessment and adjustment of the VR activities to ensure they remain relevant and motivating.

Despite the positive effects associated with measuring progress in VR, the process can be difficult [55]. This difficulty arises from the need to ensure that the metrics used in VR accurately reflect real-world abilities and improvements. While many VR games are primarily designed for entertainment rather than rehabilitation, the importance of involving users in the co-design of VR applications is increasingly recognized [25].

The use of VR in this study revealed that it is beneficial as a complementary tool in stroke rehabilitation, enhancing user engagement, increasing training intensity, and providing structured exercises in both clinical and home environments. While VR cannot replace traditional rehabilitation methods, it can serve as a powerful addition that supplements existing therapy approaches. Previous studies suggest that integrating specific VR technologies with traditional rehabilitation approaches can lead to greater improvements in motor function and activity levels in people after stroke compared with using conventional rehabilitation methods alone [56-58]. For example, VR can provide repetitive and varied exercises that target specific motor skills, helping people after stroke to practice and refine their movements in a controlled environment, thereby freeing therapists from the mundane tasks of high-volume training.

A potential benefit could also be, as found in a study [59], that VR demonstrated significantly greater therapeutic effects than conventional training in improving upper limb function, as assessed by the Fugl-Meyer and Action Arm Research Tests [32]. Following the stroke trajectory, VR could be implemented during the hospital stay, as a prepractice and supplement to real-life activities. In the home setting, it would need to be scaled down to accommodate safety precautions, but has the possibility to be further scaffolded when returning for follow-up at the hospital in cooperation with health care professionals. A significant challenge in using VR for rehabilitation is ensuring its effective integration with traditional therapy while maintaining professional oversight as rehabilitation extends beyond clinical settings.

Successful integration is essential, as it necessitates a seamless combination of VR-based and conventional approaches to optimize therapeutic outcomes. Professional oversight remains crucial for monitoring patient progress, adjusting interventions, and providing ongoing support tasks that become increasingly difficult to maintain once people transition to home-based rehabilitation after stroke.

### Implications for Practice

Our findings suggest that VR could be a valuable supplement to traditional rehabilitation for specific stroke groups, but it cannot replace the essential contact with the therapist, clinical reasoning, or the assessment of activity limitations and opportunities. We propose that VR could be particularly beneficial for a younger population with stroke who lack sufficient access to home-based rehabilitation and struggle with motivation for self-training. Additionally, VR may help maintain function in individuals in the chronic phase after a stroke, where repetition and high-quality training are crucial to prevent functional decline. However, it is important to acknowledge that VR cannot fully replicate training in real-life, everyday activities. For VR solutions to be effective, they must be carefully tailored to the individual’s functional level and ease of use. This tailoring process, however, can be resource-intensive in an already strained health care system.

In light of this, we raise important questions about who will truly benefit from VR, given the wide range of outcomes following a stroke, and whether, in some cases, it may be more appropriate to prioritize traditional therapy. Finally, we emphasize the importance of training for relatives and caregivers, as their role may be critical to the successful use of VR at home.

### Limitations and Future Work

The limitations of this study primarily stem from the use of a qualitative single case study design [27], which provides an in-depth understanding of the real-life phenomenon but may limit the generalizability of the findings across broader contexts. The reliance on rapid prototyping and TA, while effective for capturing detailed insights into VR usage in poststroke rehabilitation, may not fully address the variability of experiences and needs among a wider population of people after stroke. Also, the rapid evolution of VR technology means that some of the results will soon be outdated, limiting the applicability of the findings to current and future developments in the field [55,60].

Future work should aim to expand the scope of research by incorporating multiple case studies across diverse settings to enhance the generalizability of the results. This could involve a larger and more varied participant pool to capture a broader range of experiences and needs. Future work should focus on developing integrated, individualized therapy solutions that seamlessly combine arm therapy, cognitive therapy, and mental support within a single solution, addressing the multifaceted rehabilitation needs, such as cognitive needs or physical therapy needs, of individuals with stroke as described in Gkintoni et al [61].

This requires implementing design methodologies that facilitate co-creation with diverse stakeholders, including health care professionals, VR developers, and people after stroke with varying needs, ensuring that the solutions are both inclusive and effective. Efforts should also be directed toward creating a smooth transition from hospital settings to home environments for people after stroke, as described by Pourliaka et al [62], using VR to extend rehabilitation beyond clinical settings while maintaining professional oversight.

Furthermore, the development of AI-powered solutions that account for the personalized goals, needs, and situations of people after stroke is crucial, enabling tailored interventions that adapt to individual progress and challenges. For instance, the system could select the most appropriate exercises based on how individuals perform each exercise [63]. One option is to incorporate validated assessments, such as the short-form of MoCA [31,64], to evaluate patient situations accurately and guide therapy adjustments.

Designing solutions that not only excite individuals with stroke, but also genuinely facilitate improvements in their therapy goals and daily life challenges is vital. This involves crafting engaging VR environments that motivate consistent participation while ensuring real-world relevance and measurable benefits in rehabilitation outcomes. Further exploration of potential scenarios that can adapt to the individual requirements of home rehabilitation over time is also essential.

Additionally, more validation studies, for example, pilot trials or feasibility studies to evaluate the preliminary effects and randomized controlled trials to evaluate actual VR solution outcomes, are urgently needed [54]. In addition, standardized usability and comfort measures, such as the System Usability Scale and the Virtual Reality Sickness Questionnaire, were not used. Their inclusion could have offered more structured and quantifiable assessments of user experience, particularly regarding interface usability and potential adverse effects like motion sickness. Future work should consider integrating these validated instruments to complement qualitative feedback and provide a more comprehensive evaluation of user interaction and comfort.

### Conclusion

This study underscores the transformative potential of VR in advancing stroke rehabilitation. VR provides a dynamic and interactive platform that can be customized to support the individualized trajectories of recovery, accommodating the physical limitations, cognitive profile, and personal goals of people after stroke. Its core strength lies in its adaptability by offering immersive simulations of real-world tasks that foster motivation and engagement through meaningful and goal-directed experiences. However, the successful application of VR in rehabilitation requires more than technological advancement. It needs to be integrated thoughtfully with established rehabilitation principles, including task-specific training, measurable and ecologically valid outcomes, as well as alignment with the evolving needs of individuals. A critical insight from this study is the necessity of embedding clear therapeutic intent into VR interventions. Whether the objective is motor recovery, cognitive enhancement, or balance restoration, each activity should serve a purpose that is clinically relevant and meaningful to the user’s daily life. This demands a comprehensive understanding of individual motivations, challenges, and aspirations through involving people after stroke in the design process. It is through participatory co-design that developers can ensure that VR experiences are both engaging and clinically appropriate. As an emerging technology, VR should not be considered a replacement for traditional therapy, but rather a powerful adjunct that can increase training intensity, reduce the burden on therapists, and extend rehabilitation into home settings, making therapy more accessible and continuous. When thoughtfully designed and implemented, VR can bridge the gap between structured clinical care and independent recovery, providing consistent support throughout the rehabilitation journey.

## Appendix

Multimedia Appendix 1 Interview and workshop guide.

## Abbreviations

AI: artificial intelligence
MoCA: Montreal Cognitive Assessment
TA: thematic analysis
VR: virtual reality

## Data Availability

The dataset is derived from interviews in Norwegian and is not publicly available due to consent restrictions and data collection approval. Parts of the data can be made available from the corresponding author upon request.
