# Supplementary material for: Challenges and Benefits of Virtual Reality in Home-Based Poststroke Rehabilitation: Co-Design Qualitative Study
Source: JMIR Hum Factors. 2026 Jan 15;13:e78798. doi: 10.2196/78798 (PMC12856395; doi:10.2196/78798)
Supplement: Multimedia Appendix 1 [file humanfactors_v13i1e78798_app1.docx]

**INTERVIEW GUIDE FOR HEALTH CARE PROFESSIONALS AND VR TECHNOLOGY DEVELOPERS + WORKSHOP GUIDE**

**INTERVIEW WITH HEALTHCARE PROFESSIONALS**

**Background information on needs and challenges of stroke rehabilitation**

- Can you describe the current rehabilitation process for stroke patients after they are discharged to continue recovery at home?
- How are rehabilitation goals typically set, monitored, and adjusted for stroke patients recovering at home?
- What aspects of your daily work with stroke patients at home are the most resource-intensive or time-consuming?
- From your perspective, what are the biggest challenges stroke patients face during home-based recovery?

**Current use of VR technologies to help stroke patients**

- Explain how you have used VR technologies for rehabilitation?
- What was it like preparing equipment?
- Did you find the VR technologies useful in the rehabilitation of post stroke survivors?
- What are the advantages/disadvantages you personally see?
- What do you think about using individual vs. group therapy for stroke home rehabilitation?
- Are there features of the VR technology you see as good or problematic especially when patients are alone at home? Reflect on the 3 scenarios presented in the use of VR technologies.
- What specific adaptations should a VR system have to support patients with varying levels of physical ability?
- What kind of capabilities might the solution have to support patient navigation?

**Ways to improve the effectiveness of VR technology in rehabilitation**

- What changes or improvements would make VR technologies more practical and effective for home-based use?
- What have you found most helpful or effective when using VR rehabilitation tools with stroke patients?
- What challenges or limitations have you encountered when using VR rehabilitation tools, either for yourself or for your patients?
- From your perspective, what are the ways VR rehabilitation tools could be improved?

**INTERVIEW WITH THE VR TECHNOLOGY DEVELOPERS**

**Design Challenges & Constraints**

- What are the main cognitive or physical limitations which you consider when designing VR for post-stroke patients?
- What do stroke patients typically find difficult when learning or using new technologies?
- What kind of guidance or feedback do patients need while using rehabilitation tools independently?
- What are the biggest design or technical challenges you have encountered when creating VR solutions for healthcare or rehabilitation?
- How do you manage the trade-off between immersive interaction and ease of use, particularly for users with physical limitations.
- What design considerations do you take into account to make VR setups usable in a home environment?
- How do you ensure the safety and comfort of users who might use the system unsupervised?

**Features, Functions and Interaction Models**

- How do you design feedback systems in VR to guide or motivate users without overwhelming them?
- Are there specific gamification elements or adaptive features that you have found particularly effective or difficult to implement?
- Do your systems support customization or personalization based on individual user needs or therapy goals?

**Deployment & Accessibility**

- What limitations currently affect your ability to deliver scalable, home-friendly VR solutions?
- How do you balance performance and affordability in the design of VR hardware for clinical use?
- What steps do you take to simplify installation, setup, and onboarding for non-technical users?

**Innovation, Roadmap & Future Outlook**

- Where do you see the biggest opportunities for innovation in VR rehabilitation over the next 3–5 years?
- Are there features or technologies you are excited about but have not yet implemented due to technical or regulatory barriers?
- How do you see your role in supporting long-term engagement and rehabilitation outcomes, even if you are not directly involved in clinical testing?

**WORKSHOP GUIDE WITH POST-STROKE SURVIVORS**

**Can you tell us about your experiences when testing the digital solutions?**

- How easy or difficult was it to use the VR solution, based on your overall impression?
- Did the VR experience feel engaging, inviting, or interesting to you? Why or why not?
- Did the solution perform in the way you expected? Why or why not?
- Was there anything you thought the solution would include that was missing? Or anything that appeared differently than expected?
- Were there any surprises while using the solution—positive or negative? If so, what were they?
- Were there any features you found particularly useful or would like to use more often?
- Were there features you didn’t use much or found less helpful? Why?
- Were there any situations where the solution felt especially useful—or not useful at all? Can you describe those?
- Did anything about the usage scenario feel confusing, difficult, or out of place? What stood out to you?
- Was there anything in the design or experience that felt unnecessary, awkward, or misplaced? If so, what would you change?
- How would you rate the difficulty of using this VR solution overall?
- On a scale from 0 to 10, how difficult did you find this specific task or interaction?
- How did using VR impact your experience of the rehabilitation task—positively or negatively?

**What have you learned, what would you change?**

- If you could change anything about this technology, what would you improve or do differently?
- What kind of changes or improvements do you expect to see as a result of using this solution in rehabilitation?
- What have you learned from using this solution in a rehabilitation setting?
- Were there any features you found especially helpful or effective?
- Have there been situations where the solution did not contribute to patient progress? Can you describe what happened?
